# Migration of a Surgical Clip Into the Common Bile Duct and Its Spontaneous Passage

**DOI:** 10.7759/cureus.76275

**Published:** 2024-12-23

**Authors:** Zachary K Woodward, Goutham Sivasuthan, Chee Hua Lim, Ratna Aseervatham

**Affiliations:** 1 General Surgery, Sunshine Coast University Hospital, Birtinya, AUS; 2 Gastroenterology, Sunshine Coast University Hospital, Birtinya, AUS

**Keywords:** biliary obstruction, common bile duct, conservative surgical management, laparoscopic cholecystectomy, surgical clip migration

## Abstract

Cholecystectomy is one of the most commonly performed surgical operations worldwide. A rare complication following this procedure is the migration of surgical clips used to secure the cystic duct and artery. Herein, we report the migration of a metallic surgical clip into the common bile duct of a 75-year-old gentleman who underwent a laparoscopic cholecystectomy 24 years prior. He presented to the hospital three times over the course of six months with the predominant symptoms of right upper quadrant pain. His symptoms improved with supportive care during his first two admissions, and he was discharged home with a plan for ongoing investigation. Subsequent endoscopic ultrasound and magnetic resonance imaging did not identify a cause for his symptoms. On his third presentation to the hospital, he had mild transaminitis with elevated bilirubin and his computed tomography scan revealed migration of a surgical clip into the distal common bile duct where it was causing obstruction. As he was also symptomatic with influenza A and at a heightened anaesthetic risk, a conservative management approach was taken instead of upfront endoscopic retrograde cholangiopancreatography for clip retrieval. Over the coming days, his bilirubin and liver function tests began to normalise and the offending clip was not identified on a repeat computed tomography scan performed three days later, indicating spontaneous passage of the clip from the common bile duct. On re-review of the initial imaging, the surgical clip can be seen within the remnant cystic duct prior to its migration into the distal common bile duct. Surgical clip migration is an exceedingly uncommon occurrence, and this case highlights the difficulty of establishing the diagnosis despite extensive investigation. Increased awareness of this phenomenon among clinicians will hopefully aid in earlier diagnosis and improved outcomes for patients.

## Introduction

Approximately 6% of the population worldwide has gallstones, with incidence varying depending on the geographical location, female gender, age, and obesity [1,2]. Each year between 0.1% and 4.0% of people with asymptomatic gallstones will go on to develop symptomatic gallstone disease [3]. The gold standard treatment for symptomatic gallstone disease is laparoscopic cholecystectomy and remains one of the most commonly performed surgical procedures. In Australia, the rate of cholecystectomy is 197 per 100,000 people [4].

During a cholecystectomy, the cystic artery and duct are typically secured with either metallic or polymer surgical clips. Postoperative complications following the surgery include bleeding, bile duct injury, and biliary leak. A comparatively rare complication is the migration of surgical clips into other structures, including the common bile duct. Surgical clips that have migrated into the common bile duct can cause biliary obstruction, cholangitis, and pancreatitis and can also act as a nidus for the formation of cholelithiasis [5,6].

Herein we report a patient who had symptomatic migration of a metallic surgical clip into the common bile duct 24 years after a laparoscopic cholecystectomy and the subsequent spontaneous passage of the clip.

## Case presentation

A 75-year-old gentleman presented to the hospital three times over a six-month period. His relevant past medical history included atrial fibrillation, gastroesophageal reflux disease, depression, a lumbar fusion, and an uncomplicated laparoscopic cholecystectomy performed 24 years prior.

On his first presentation, his chief complaint was right upper quadrant pain which had been gradually worsening over the last three days; he denied any similar episodes of pain previously. His blood analysis showed a normal white cell count with a mildly elevated C-reactive protein (CRP) to 31 mg/L (reference range: <5.0 mg/L). His liver function tests were also mildly elevated alkaline phosphatase (ALP) 146 U/L (reference range: 30-110 U/L), gamma-glutamyl transferase (GGT) 263 U/L (reference range: <55 U/L), alanine transaminase (ALT) 242 U/L (reference range: <45 U/L), and aspartate aminotransferase (reference range: AST) 74 U/L (reference range: <35 U/L) with a normal bilirubin. A computed tomography (CT) scan showed intra- and extra-hepatic duct dilatation with no evidence of a filling defect within the common bile duct. Ultrasound showed a small echogenic focus in the distal common bile duct concerning for an intraductal calculus; however, no choledocholithiasis was seen on subsequent magnetic resonance imaging (MRI) scan. After two days in the hospital, the patient's pain improved, his liver function tests were downtrending, and he was discharged home.

The patient subsequently re-presented a month later with similar pain which had been worsening over a week. At this time, his blood analysis showed no abnormality except a raised CRP of 99 mg/L. A CT scan revealed an inflammatory change surrounding the remnant cystic duct raising the possibility of cholangitis; however, he remained afebrile and clinically well. A repeat MRI did not show any change from the previous scan. He received antibiotics in the hospital for three days with improvement in his pain and was discharged home. For further investigation, the patient underwent an endoscopic ultrasound as an outpatient; this again showed a dilated bile duct with no cholelithiasis or biliary sludge.

**Figure 1:**
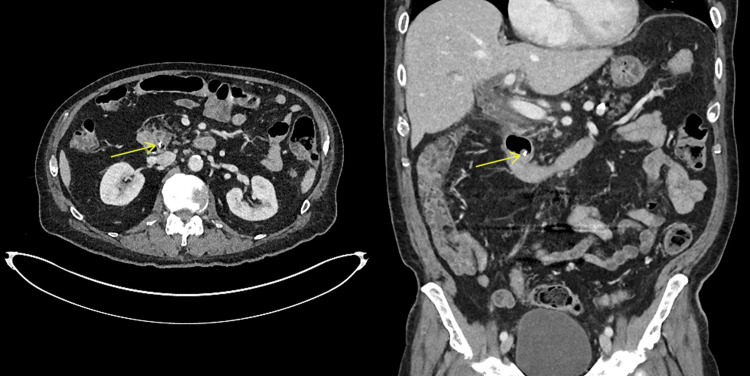
Axial and coronal CT images from the patient's third presentation to the hospital showing the migrated surgical clip in the distal common bile duct causing biliary obstruction.

Four months later, the patient re-presented to the hospital with similar ongoing pain, nausea and vomiting, fevers, and coryzal symptoms. His blood analysis showed a mild transaminitis with ALP 200 U/L, GGT 591 U/L, ALT 154 U/L, AST 84 U/L, and an elevated bilirubin of 72 umol/L (reference range: <20 umol/L). His white cell count was normal with a raised CRP of 278 mg/L; however, he also tested positive for influenza A. A CT scan revealed that a metal cholecystectomy clip had migrated from the remnant cystic duct into the distal common bile duct and was causing obstruction (Figure 1).

Due to periprocedural anaesthetic risks from intercurrent influenza infection, a conservative management approach was adopted instead of upfront endoscopic retrograde cholangiopancreatography for clip retrieval. Over the following days, his pain resolved, and his bilirubin and liver function tests normalised. A repeat CT scan three days after presentation showed improvement of the biliary dilatation and no sign of the offending surgical clip, indicating spontaneous passage of the clip from the common bile duct.

**Figure 2:**
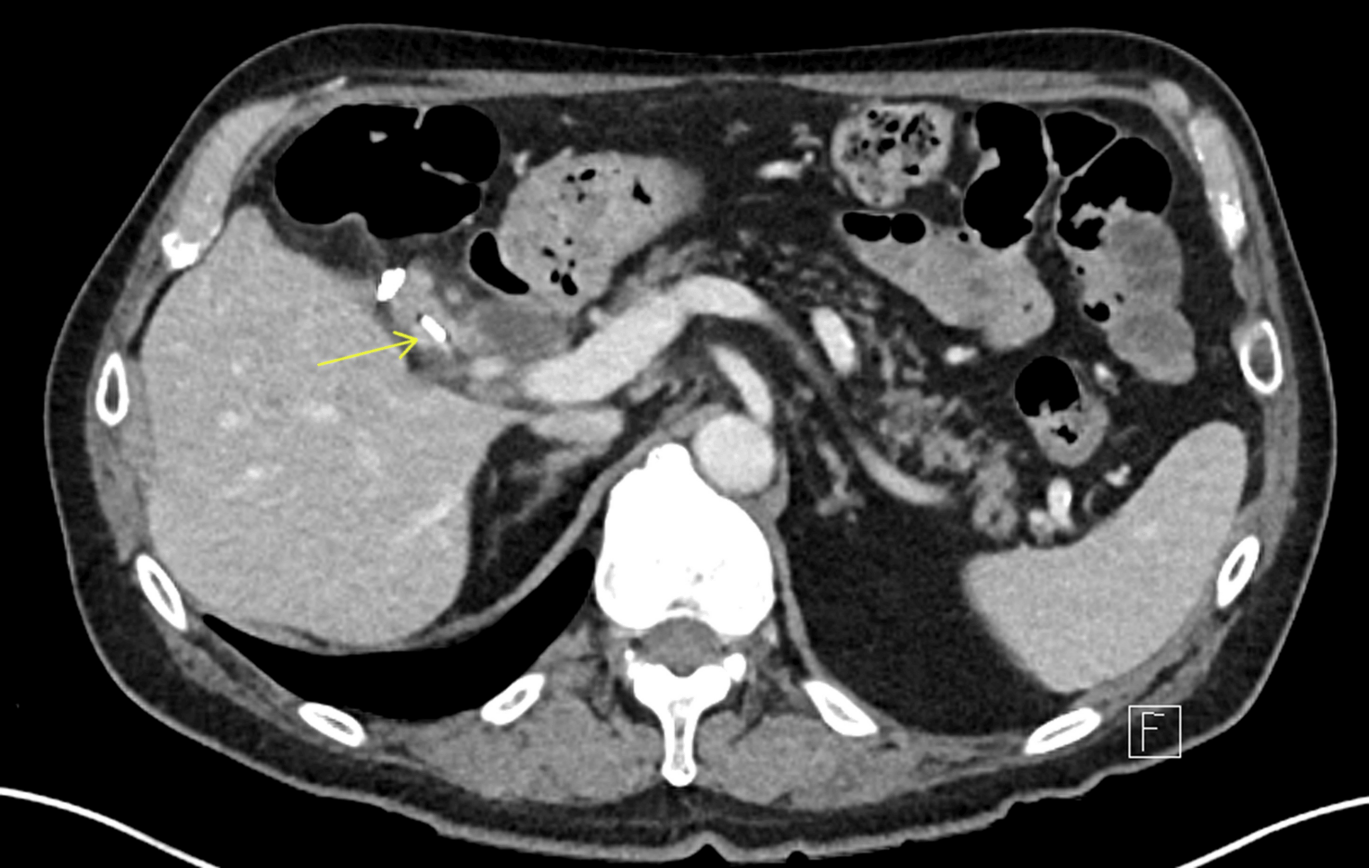
Axial CT from the patient's second presentation to the hospital showing migration of the surgical clip to the remnant cystic duct. Inflammatory stranding can be seen surrounding the migrated clip.

**Figure 3:**
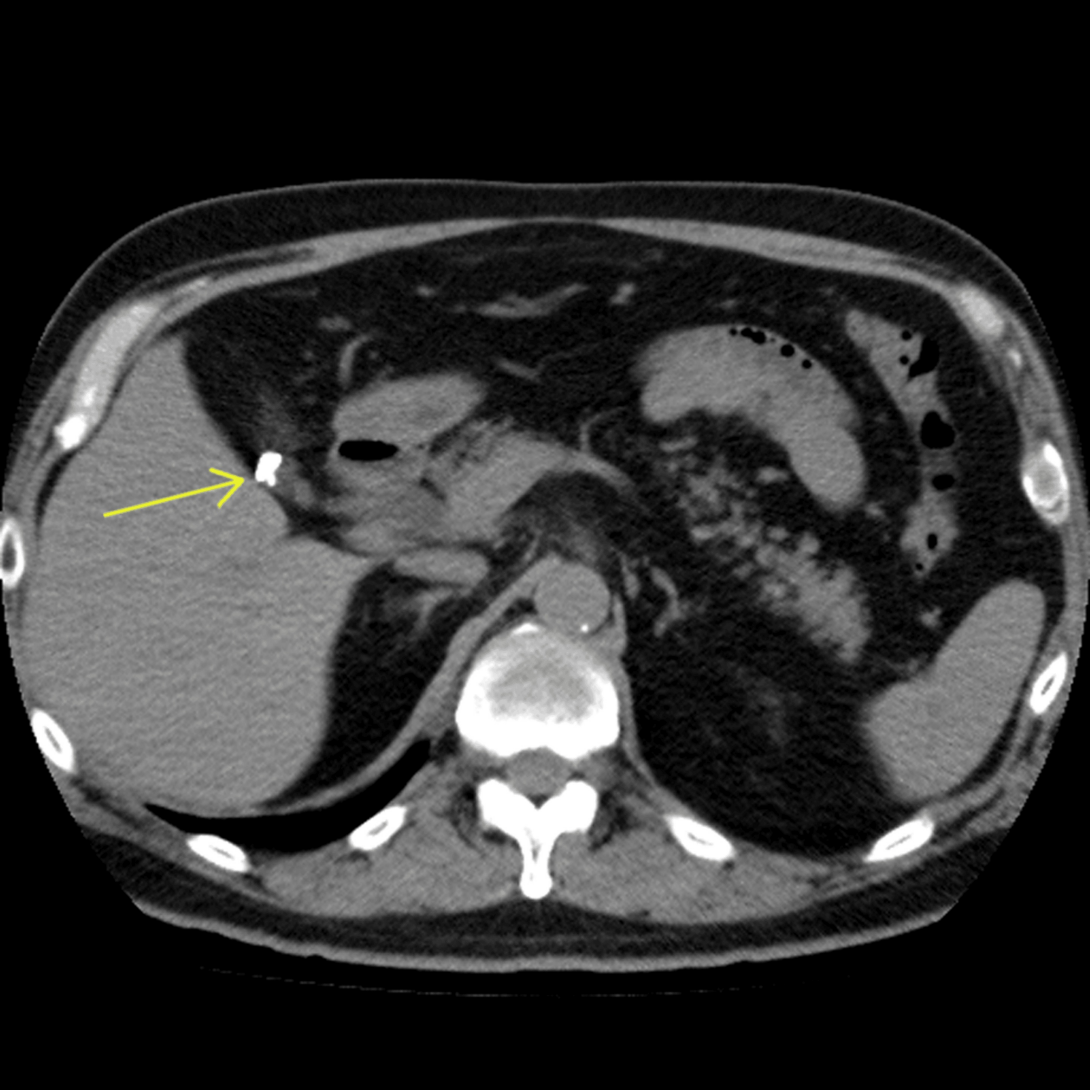
Axial CT performed 15 years prior to the patient's development of symptoms showing no evidence of clip migration and the correct placement of all surgical clips.

On re-review of the patient's CT images from the previous admissions, a surgical clip can be seen within the lumen of the remnant cystic duct (Figure 2); however, this went unrecognised at the time. To better understand when the migration of this surgical clip occurred, additional past imaging from external providers was sought for comparison. A CT scan from 15 years prior was identified which showed that all surgical clips were correctly positioned (Figure 3), indicating that the clip migration occurred within this timeframe. On follow-up after three months, the patient remains well and has been asymptomatic.

## Discussion

Surgical clip migration is a rare but clinically important complication following cholecystectomy. Clip migration into the common bile duct may present with pain, biliary obstruction, cholangitis, or pancreatitis [6]. Migrated clips have also been reported to act as a nidus for the formation of cholelithiasis [5].

The exact pathological mechanism responsible for surgical clip migration remains unknown, and the specific causative factors contributing to clip migration may vary depending on the case under scrutiny. Factors that are expected to contribute to clip migration include cholecystectomies complicated by inaccurately placed clips, ineffective closure of the bile duct, bile duct injuries, bile leak, use of multiple surgical clips, and local inflammation with or without infection [6,7]. Once a clip becomes lodged within the bile duct wall, it can subsequently migrate through the biliary wall and into the duct, following the path of least resistance. Additionally, it has been hypothesised that local compression of the remnant cystic duct may cause invagination of the cystic stump and clip, which facilitates its migration into the bile duct [7,8].

The median interval between cholecystectomy and clinical presentation of symptoms is 24-49 months with a range of 11 days up to 35 years [6,7]. The factors in this case which precipitated the migration of the surgical clip after such a lengthy delay remain unclear. The patient also denied any obvious inciting factors such as a history of trauma.

The most common treatment for retained biliary clips with or without stone formation is retrieval with endoscopic retrograde cholangiopancreatography which has a success rate of between 54 and 77% [7]. However, cholangioscopy and surgical retrieval have also been reported in the literature as alternative options for clip removal [9,10]. In the presented case, the clip underwent spontaneous passage from the common bile duct after a period of watchful waiting, suggesting that a conservative approach to management may be preferable in some patients as the risks associated with endoscopic or surgical retrieval can be avoided. However, it is difficult to predict which patients may benefit from this approach as there are only a few reports of spontaneous clip passage in the literature [6,11,12].

Biliary migration appears possible with almost any foreign material including plastic clips, suture material, and absorbable surgical clips [6]. Although these non-metallic materials are more difficult to diagnose radiologically, both CT and MRI have been utilised to detect the migrated material itself, or the resulting stone which can form around the migrated object [13,14]. To prevent this complication, clipless cholecystectomy using an ultrasound-activated harmonic scalpel has been shown to be a safe alternative to conventional cholecystectomy and does not leave any foreign in situ which has the potential to migrate [6]. However, the cost of the instrument may be a limitation.

This case also illustrates the difficulty with establishing this diagnosis as despite extensive investigation with imaging; surgical clip displacement was not considered until the clip had migrated into the distal common bile duct. Additionally, if the patient's previous imaging was available for comparison, the migrated surgical clip may have been detected earlier. With hindsight, the patient’s initial symptoms were likely the result of the surgical clip causing a localised biliary obstruction and cholangitis-like effect within the remnant cystic duct.

## Conclusions

Migration of surgical clips into the common bile duct is a clinically important complication that can occur many years after cholecystectomy. Although rare, we believe if there were a greater awareness of this phenomenon, then perhaps the diagnosis could have been made earlier. Migrated surgical clips should be considered as a potential differential diagnosis for patients with previous cholecystectomy presenting with symptoms of choledocholithiasis or cholangitis. In select patients, a trial of conservative management may allow for spontaneous passage of retained clips from the common bile duct.
